# Speech-based characterization of dopamine replacement therapy in people with Parkinson’s disease

**DOI:** 10.1038/s41531-020-0113-5

**Published:** 2020-06-12

**Authors:** R. Norel, C. Agurto, S. Heisig, J. J. Rice, H. Zhang, R. Ostrand, P. W. Wacnik, B. K. Ho, V. L. Ramos, G. A. Cecchi

**Affiliations:** 1 IBM T.J. Watson Research Center, Yorktown Heights, NY 10598 USA; 2 Pfizer Digital Medicine & Translational Imaging: Early Clinical Development, Cambridge, MA 02139 USA; 3 Department of Neurology, Tufts University School of Medicine and Tufts Medical Center, 800 Washington St, Boston, MA 02111 USA

**Keywords:** Signs and symptoms, Neurology

## Abstract

People with Parkinson’s (PWP) disease are under constant tension with respect to their dopamine replacement therapy (DRT) regimen. Waiting too long between doses results in more prominent symptoms, loss of motor function, and greater risk of falling per step. Shortened pill cycles can lead to accelerated habituation and faster development of disabling dyskinesias. The Unified Parkinson’s Disease Rating Scale (MDS-UPDRS) is the gold standard for monitoring Parkinson’s disease progression but requires a neurologist to administer and therefore is not an ideal instrument to continuously evaluate short-term disease fluctuations. We investigated the feasibility of using speech to detect changes in medication states, based on expectations of subtle changes in voice and content related to dopaminergic levels. We calculated acoustic and prosodic features for three speech tasks (picture description, reverse counting, and diadochokinetic rate) for 25 PWP, each evaluated “ON” and “OFF” DRT. Additionally, we generated semantic features for the picture description task. Classification of ON/OFF medication states using features generated from picture description, reverse counting and diadochokinetic rate tasks resulted in cross-validated accuracy rates of 0.89, 0.84, and 0.60, respectively. The most discriminating task was picture description which provided evidence that participants are more likely to use action words in ON than in OFF state. We also found that speech tempo was modified by DRT. Our results suggest that automatic speech assessment can capture changes associated with the DRT cycle. Given the ease of acquiring speech data, this method shows promise to remotely monitor DRT effects.

## Introduction

Parkinson’s disease (PD) is the second most common neurodegenerative disease, with an estimated prevalence of 0.3% in industrialized countries, 1.0% in people over 60, and 3.0% in people over 80^1^. Roughly 10 million people worldwide live with PD, and ~60,000 Americans are diagnosed with PD each year^2^. Balance and gait disturbances in people with Parkinson’s (PWP) lead to falls, mobility loss, serious injuries, and reduced independence^3,4^. Close to 90% of PWP develop speech disorders, leading to significant decline in quality of life due to substantial deterioration in functional communication^5,6^.

At present, the most common treatments for PD contain L-DOPA and there is evidence that dopamine replacement therapy (DRT) improves functional balance^4,7^. Unfortunately, prolonged use of DRT often results in habituation, leading to reduced symptom control^8^, fluctuations of symptom relief known as “ON” (symptom relief) and “OFF” (reduced symptom relief) states^9,10^, and dyskinesias. To characterize the progression of PD, the most widely-used clinical rating scale is the Unified Parkinson’s Disease Rating Scale (MDS-UPDRS)^11^ of which part III characterizes motor activities. The MDS-UPDRS was designed for occasional in-clinic evaluation, rather than frequent monitoring. Administering the rating scale requires training and certification from the Movement Disorder Society. The MDS-UPDRS part III includes a section for scoring speech in five levels; 0: normal (no problems); 1: slight (speech is soft, slurred or uneven); 2: mild (occasionally parts of the speech are unintelligible); 3: moderate (frequently parts of the speech are unintelligible); and 4: severe (speech cannot be understood). It has been noted that the inter-rater reliability of speech scores on the UPDRS (rather than the MDS-UPDRS) is inconsistent^12,13^, and recently Nöth et al.^14^ showed that the MDS-UPDRS does not accurately capture deterioration in communication. The current gold standard for at-home continuous (every half hour) recording of a patient’s “ON” and “OFF” state is the self-reported Hauser^15^ diary. This technique places the burden of monitoring on the patient and the results may be confounded by other factors, such as the patient’s mood and lack of sleep, and definitionally relies on the patient’s subjective self-assessment. Additionally, differences in self-assessment and objective assessment of speech deficiencies in PWP have been reported, probably due to adaptation to changes^16^. For these reasons, alternative methods reducing the burden on patients and at the same time providing consistent assessments of medication state at home are of great importance for the PD community. These methods would not only infer current medication state, but also address a currently unmet need for the information necessary to determine optimal pill cycle timing on an individual level.

Speech has been shown to be different between PWP and controls^17–19^ and to be affected by dopamine levels in PWP^20^. Features of speech applicable to monitoring a patient’s long-term disease progression include: reduced loudness, decreased variability in pitch and intensity, reduced stress, breathiness and hoarseness, and imprecise articulation^17^. For three recent reviews of speech features applicable in monitoring PD progression see refs. ^18,19,21^. Previous studies have also shown that speech features can differentiate healthy controls from PWP, most notably by using acoustic measurements of sustained phonations^22–25^. Recently, an automatic evaluation of dysarthria in PWP was performed^26^ by analyzing six types of diadochokinetic (DDK) exercises. However, studies on the effectiveness of using changes in speech production to differentiate PWP in the “ON” versus the “OFF” states have generated mixed or contradictory results. Okada and colleagues^27^ studied vowel articulation in PWP and reported that vowel space was significantly expanded after DRT, contrary to previous findings^28^ which found no change in vowel space before and after treatment. Fabbri and colleagues analyzed a cohort of late-stage PD patients^29^ and similarly did not find significant changes in speech as assessed by clinical evaluation and automated analysis of voice stability/variability following an otherwise positive L-DOPA response. In a recent meta-analysis Pinho and colleagues^30^ report that DRT modifies F0 (fundamental frequency) and jitter, but does not have an impact on vocal intensity. Smith et al. ^18^ showed differences in PWP and aged matched controls in word-finding-difficulties by analysis of the semi-structured Cookie Theft description test. There is also evidence that cognitive impairment, which can be a prominent feature of advanced/late stage of PD, either affects or is reflected in language production^19^. Embodied cognition postulates that the motor system influences cognition. In particular, it has been suggested that action words and motor representation of those actions activate the same network in the brain^31,32^. One difference between PWP and healthy controls, and PWP in “ON” vs. “OFF” state is the use of action verbs^33–37^. These are verbs that describe actions such as “run” or “swim,” as compared with verbs that describe mental states or emotions such as “think” or “hope”. PWP typically produce fewer action verbs than healthy controls^33,38,39^.

Based on these findings, we evaluated the speech of PD participants during two medication states (ON vs. OFF) on three different speech tasks: picture description, diadochokinetic rate test, and reverse counting. These tasks were characterized with acoustic (cepstral analysis), prosodic (speech tempo), and linguistic (semantic embedding) features. In this study, we aim to test the following hypotheses: (i) DRT causes changes in speech that can be detected using are reflected in acoustic, prosodic and semantic features, which can be detected using automatic methods, (ii) tasks involving semi-structured free speech can provide more information than structured tasks to assess medication states, and (iii) the use of speech features associated with action verbs, which are relevant for the discrimination of PWP and controls, would be equally applicable for differentiating ON and OFF medication states.

## Results

### Statistical analysis

**Fig. 1:**
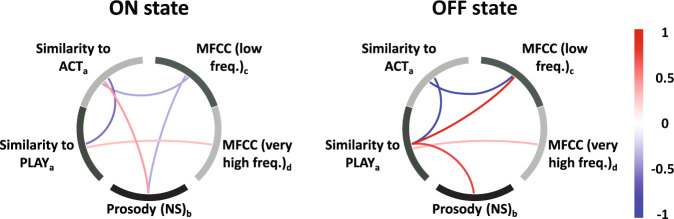
Comparison of Partial correlations for top features for both states. Partial correlations for “ON” and “OFF” states were calculated using the top five features of the picture description task, as listed in Table 1. Positive correlations are displayed in red while negative correlations are in blue. “OFF” state shows a stronger correlation among these five features in comparison with “ON” state. Notes: Sub-index in the name of the feature indicate the statistical descriptor: **a** robust minimum (computed as 10th percentile), **b** robust maximum (computed as 90th percentile), **c** mode (the most frequent value), **d** skewness (a measure of asymmetry in the distribution of values).

Table 1 shows the top 5 ON/OFF statistically significant discriminating features after applying a Bonferroni correction for multiple comparisons (α = 0.05) for each of the three speech tasks. Note that reverse counting and diadochokinetic rate are dominated by features of low-frequency energy (MFCC1) and high-frequency energy (MFCC11), respectively. High-frequency energy captures changes in perceived hoarseness^40,41^ in PWP. Features from the picture description task captured significant changes both in low and high frequency in addition to speech rate and semantic content. This conformed to the expected increased richness of its feature set. The top 5 variables were: the robust minimum (10th percentile) for the concepts of “act” and “play”, which confirmed the differential use of action verbs as a result of medication, in concordance with the reported differential use of action verbs between PWP and^33–39,42^; the mode (most frequent value) of a low-frequency MFCC spectral energy, the skewness (asymmetry) of a very high-frequency MFCC spectral energy, and the robust maximum (90th percentile) of the distribution of inter-syllable time intervals, which indicates longer tails of the distribution in ON state, suggesting more control on speech production. We evaluated patterns of co-variation among the five top-ranked features for the picture description task using partial correlation (see Fig. 1). The “OFF” state was characterized by strong positive partial correlations between SF (play) and acoustic (MFCC #2) and SF (act and play) and NS (pct90) features.

**Table 1:** Top ranked features.

Speech Task	Feature	*p*-value	*t*-statistic
Picture description	PLAY (pct10)	5.9e−07	5.76
	MFCC #2 (md)	1.5e−05	4.82
	ACT (pct10)	2.8e−05	4.63
	MFCC #12 (sk)	4.0e−04	−3.81
	NS (pct90)	5.1e−04	3.73
Reverse counting	MFCC #1 (q50)	1.5e−07	−6.14
	MFCC #1 (q25)	9.0e−07	−5.63
	MFCC #1 (mn)	4.1e−06	−5.20
	MFCC #1 (q75)	4.9e−06	−5.15
	MFCC #8 (sk)	3.19e−05	4.60
Diadochokinetic rate	MFCC #11 (pct75)	2.5e−05	4.69
	MFCC #11 (mn)	1.1e−04	4.24
	MFCC #11 (pct50)	1.9e−04	4.06
	MFCC #3 (pct75)	2.6e−03	−3.19
	MFCC #11 (pct25)	2.7e−03	3.17

The five top-ranked features for “ON” vs “OFF” states characterization for each speech task. Ranking is calculated with all of the extracted features using two-sample *t*-test; the features listed are statistically significant (*p* < 0.05) after multiple testing correction. A positive *t*-statistic indicates greater mean value for the ON state.

### Classification

**Fig. 2:**
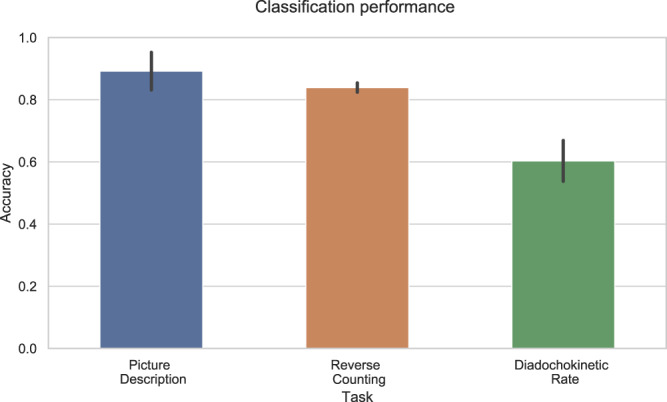
Classification accuracy. Classification performance for each task using feature selection, using the five top-ranked features using 10-fold (by subject) cross validation. Bars show mean of 50 runs, vertical lines denote standard deviation. Results surpass chance probability.

Binary classification was performed by subtracting feature values for one medication state from the other. Table 2 presents the highest accuracy achieved for each combination of acoustic, prosodic, and content features. Figure 2 shows the best performance for each of the three speech tasks, picture description, reverse counting, and diadochokinetic rate. For picture description, the combination of acoustic (MFCC), prosodic (NS) and semantic feature types resulted in a top classification accuracy of 0.89. For reverse counting, acoustic features alone provided for a classification accuracy of 0.84. Finally, for diadochokinetic rate, the best result was obtained using also acoustic features alone, resulting in an accuracy rate of 0.60.

**Table 2:** Classification performance.

Speech Task (# patients)	Features	Classifiers	Top 5 features
Picture description (25 patients)	NS	RF	0.61 ± 0.05
	SF	RF	0.77 ± 0.04
	NS + SF	EN	0.79 ± 0.07
	MFCC	EN	0.54 ± 0.08
	MFCC + NS	EN	0.50 ± 0.06
	MFCC + SF	LR-l1	0.89 ± 0.06
	MFCC + SF + NS	LR-l1	0.89 ± 0.05
Reverse counting (25 patients)	NS	RF	0.41 ± 0.07
	MFCC	NB	0.84 ± 0.02
	MFCC + NS	RF	0.79 ± 0.03
Diadochokinetic rate (24 patients)	NS	LR-l1	0.53 ± 0.06
	MFCC	NB	0.60 ± 0.07
	MFCC + NS	NB	0.58 ± 0.06

Performance achieved in each task for the different feature sets. Only the classifiers with highest accuracy value are shown. Accuracy is computed as the average (± s.d.) of 50 runs with 10-fold cross-validation. MFCC features are relevant for achieving good performance in the different speech tasks.

## Discussion

We combine acoustic, prosodic, and semantic features of speech to predict medication state in PWP. High accuracy rates (see Fig. 2) were achieved with all speech tasks, in particular for picture description (0.89) and reverse counting (0.84). Both of these tasks have a cognitive component that can be captured by our features, which suggests that cognition may be also contributing to the differentiation of ON/OFF states on top of the speech degradation. Specifically, in our analysis we found that features obtained for the picture description task (free speech) could successfully differentiate L-DOPA “ON/OFF” states. Although it has been reported that dopamine replacement does not significantly improve speech in PWP^43–46^, there is evidence that dopamine affects motor skills which affect speech production^45^. Given that neurologists usually give the same score in ON/OFF states to speech part of MDS-UPDRS, we suggest that the effect of dopamine on speech in PWD is located in features that are undetectable for human perception (e.g., high frequency content) which can be captured with our methods. In addition, the use of better recording equipment in comparison with past decades also allowed us to detect enough subtle differences to drive the classification. The low granularity of MDS-UPDRS sub-scores contributes as well to lack of differentiation on ON/OFF state scores by humans.

In particular, our results suggest that the main difference between medication states is characterized by changes in the speech energy. This speech energy variation is characteristic of hypokinetic dysarthria found in PD^47^. MFCC #11 and MFCC #12 capture high frequency information [MFCC #11: 9.5 kHz–12.6 kHz and MFCC #12: 12.6 kHz–16.7 kHz], likely perceived by listeners as the difference in hoarseness between “ON” and “OFF” states^48^. High frequency components of speech affect intelligibility^49,50^ and differentiate between patients with dysphonia and controls^48,49^. Two rat models of PD^51^ showed rats with a damped dopaminergic system had a lower maximum frequency than controls for both simple and frequency-modulated calls. The other set of important features are the tails (10^th^ percentile or robust minimum) of the distributions of words uttered by the participants related to the seed words *act* and *play*, in particular, we observe that participants show a higher robust minimum of the similarity to these concepts when they are in ON than when they are in OFF. This is consistent with the hypothesis that L-DOPA brings participants closer to a normative state, given that as already mentioned there is strong evidence of a bias against action-oriented verbs in PWP’s speech production^33–38^. To further assess the robustness of MFCCs in our analysis, we performed an experiment to evaluate the change in classification performance when the duration of the recording was reduced. The results showed that there were not significant changes when the duration of the recording was reduced down to 10 s. Our relatively high classification accuracy even with this short duration suggests brief ambient or prompted speech samples captured outside the clinic could be used to monitor PD patients. More information on the experiment can be found in the Supplementary material.

The results of our semantic analysis indicate that action verbs like *play* and *act* have a higher association (higher similarity distance) with descriptions from participants in ON-state vs. OFF-state (*t*-statistic of 5.76 and 4.63 reported in Table 1). This indicates that PWP in OFF state have more difficulty producing action verbs. This recapitulates findings in the previous work^33,38,39^ where the speech of PWP is compared with heathy controls. These studies show that PWP typically produce fewer action verbs such as “run” or “swim,” compared with verbs that describe mental states or emotions such as “think” or “hope”. Given that motor function influences cognition^31,32^ and motor responses are less affected in ON-state, we think language production is less affected than in OFF-state. We also think that while the production of action verbs is affected in PWP with respect to healthy participants there is also a difference in impairment within each PWP produced by medication state that can be captured by our analysis. We speculate that this difference may also be present between subjects. However, it would be necessary to include a larger cohort with healthy participants to perform a better assessment of language production.

In the picture description task, three types of features—acoustic, semantic, and prosodic—were informative and complementary to each other.

We showed (see Fig. 1) that a very high positive correlation between different features (red lines) occurred only in the “OFF” state between SF (*play*) and MFCC #2 and NS. This differential relationship in the two states, after combining the three categories of features, helped achieve better discrimination between the medication states. Specifically, we found an improvement of 35% with respect to using only MFCC features (see Table 2). Classification accuracy may have been enhanced compared to the non-free speech tasks since subjects can express emotions while describing the picture, captured with MFCCs^52–55^ and possibly by the differences in the NS distribution^40,41,56,57^.

The effect of DRT on speech identified by our multivariate approach is significant but evidently subtle. As per the neurologists’ assessment, 64% of the subjects presented an MDS-UPDRS speech score difference of 0, with one participant actually improving the score from the ON to the OFF state. Improvement in speech induced by DRT may only be overtly manifested longitudinally. Even so, this should result from the cumulative effect of weak positive changes. Comparative neuroanatomy studies of songbirds suggest a possible mechanism for these effects, based on the significant homology between cortico-basal-thalamo-cortical loops in humans and pallial (i.e. cortex-like) loops in songbirds responsible for speech and song production, respectively^45^. Dopaminergic activity is involved in song production both in its conspecific-directed and undirected forms, with the former interpretable as communication. Auditory feedback is required for learning song in juveniles^58,59^; moreover, dopamine neurons encode the error between expected performance and auditory feedback during singing, suggesting that dopamine signaling underlies song stability even in adults. To the extent that the homology is valid, a plausible hypothesis is that dopamine is also involved in maintaining speech stability through feedback monitoring^60^, so that replacement therapy may induce subtle effects. Therefore, the neurologists’ limitations to detect medication-state changes in speech may be due to the coarse-grained nature of the score categories, or the inability of human raters to distinguish differences in speech with the current assessment protocol.

Finally, we would like to mention that the present method meets the need for a quantitative metric to monitor patients’ speech, and potentially correlates with disease progression and the effect of dopamine replacement therapy. In addition, objective mathematical and computational analysis of speech can increase the granularity in the assessment and avoid human biases that result in inconsistencies between raters^12,13^. It has previously been documented that perceptual analysis of speech on PD is outperformed by acoustic analysis^61,62^. An objective and easy method to monitor disease progression can have important effects on research in at least two ways. First, continuous monitoring can help gain insights into the progression of the disease and identify factors contributing to the stabilization of prognosis such as medicines, other types of treatments, and interventions. Second, when testing a new treatment in a clinical trial, continuous, unbiased monitoring is more powerful than self-reporting with all its associated biases and burden on the participant.

We combine acoustic and semantic features of speech to characterize PD medication state. Our study explored different, easily implementable speech tasks for monitoring PWP, and obtained high accuracy rates for differentiating medication states. Our best results were obtained using the picture description task, which collected participants’ free speech. Our accuracy results in this preliminary study, which ranged from 0.60 to 0.89, demonstrate the feasibility of this method to monitor PD patients and assess dopamine replacement therapy effects. These results support the potential of this approach to be used as a complementary tool to aid neurologists in monitoring PD patients. We acknowledge that one of the main limitations in this study is the small cohort of PD participants and that a larger cohort (with participant enrollment at different sites) is necessary to further validate the approach. In addition, the effects seen in this study will be best validated with participant groups which include greater variability among MDS-UPDRS speech scores as well as greater differences in overall MDS-UPDRS scores between medication states. This type of analysis is only expected to work in persons still able to communicate and understand directions. The onset of PD-related dementia or even mild cognitive impairment would be expected to impact the results. An important limitation of the current method is that the two states we characterized were pre-determined – one being “ON” and the other being “OFF”. Based on prior clinical literature, we expected there would be a change in speech quality between the two states and our method was set up to differentiate these states. When applied to two unknown states for an individual PWP, this expectation does not necessarily hold true, and a threshold for determining “no change” needs to be designed/produced to avoid finding artifactual “change” between two equivalent states. Conversely, the smaller this threshold can be, the more sensitive this method can be used to quantify changes between the two states in question. Finally, there is a continuum of states in the transition between ON and OFF that would need to be classified. Further work is necessary to extend this work to automated speech assessment in continuous, minimally obtrusive, remote patient monitoring in PWP.

## Methods

### Participants

Twenty-five participants (6 females age 67 ± 6 years; 19 males age 69 ± 7.5 years) with idiopathic Parkinson’s disease were enrolled. The average disease duration was 5.8 + 3 years. All participants provided written informed consent to take part in the study. They were the first cohort recruited in a larger study for Project BlueSky (Pfizer-IBM Research collaboration)^63^. Participants were recruited and the protocol was run at Tufts Medical Center, Boston, Massachusetts. The study was approved by the Tufts Health Sciences Campus Institutional Review Board, IRB # 12371. Inclusion criteria consisted of response to L-DOPA treatment, ability to recognize “wearing off” symptoms, participant confirmation of improvement after L-DOPA dose, and assessment of stage 3 or lower on the Hoehn and Yahr scale. Exclusion criteria were a current history of neurological disease besides PD, psychiatric illness that would interfere with participation, treatment with an investigational drug within 30 days or 5 half- lives (whichever is longer) preceding the enrollment in this study, alcohol consumption exceeding 7 drinks/week for females or 14 drinks/week for males, and use of a cardiac pacemaker, electronic pump or any other implanted medical devices (including deep brain stimulation devices). Each participant was evaluated by one of two neurologists during each visit, using the MDS-UPDRS-III protocol. PD participants were diagnosed in stages 1 (*N* = 2), 2 (*N* = 22), or 3 (*N* = 1) of the Hoehn and Yahr scale. UPDRS total scores were 35.6 ± 14.6 and 48.6 ± 13.6 for ON and OFF state respectively. Table 3 summarizes demographic information, clinical variables, and the improvement of several symptoms after L-DOPA intake for the analyzed subjects. In addition, Supplementary Table 1 provides the list of current medications for each participant.

**Table 3:** Subjects demographics.

	Category	PD participants
Demographics	Number of participants	25
	Age	68.7 ± 7.1 years
	Gender (% male)	76%
	Height	172.8 ± 6.9 cm
	Weight	88.7 ± 22.6 Kg
	BMI	28.9 ± 8.1
	Highest Education level	20% high school, 40% college, 40% post-graduate
	Dominant Hand (% right)	84%
	Native language*	96% English, 4% Chinese
Clinical Variables	Disease duration	5.8 ± 3 years
	Daily levodopa dose	368.4 ± 308.2 mg
	MoCA	26.8 ± 2.5
	Hoen and Yahr scale	2 ± 0.4
	UPDRS part III (ON/OFF)	35.6 ± 14.6/48.6 ± 13.6
	UPDRS speech (ON/OFF)	0.9 ± 0.7/1.2 + 0.6
	Affected side (right/left/both)	44%/44%/12%
L-DOPA effects (total cases/number of cases reported improvement)	Tremor	25/24
	Slowness in movement	23/21
	Mood	11/6
	Stiffness	18/14
	Pain	15/7
	Dexterity	23/20
	Cloudy mind	16/12
	Anxiety	14/5
	Muscle cramping	16/11

Summary of demographics, clinical variables, and dopamine effects of all PD patients analyzed in this work.

*One subject was not a native English speaker; however that subject has been speaking English for 37 years.

### Design and protocol

This paper describes the analysis of three speech tasks from the “Observational Study in Parkinson’s Patient Volunteers to Characterize Digital Signatures Associated with Motor Portion of the MDS-UPDRS, Daily Living Activities and Speech” conducted at Tufts Medical Center. The speech tasks were performed by each participant at each of two sessions, one before and one after L-DOPA administration, to capture behavior in the “OFF” and “ON” medication states respectively. The order of medication state per session was randomized to counterbalance possible practice effects. As a result, 13 participants were in the “ON” state for session 1 and “OFF” state for session 2, and 12 participants were in the “OFF” state for session 1 and “ON” state for session 2. To ensure that we correctly acquired the data to reflect both medication states, medication dosage and timing was dictated by the participant’s normal daily regimen of dopamine replacement therapy. Following IRB guidelines, we did not have participants take any other dosage. Both medication states were self-reported by the patients and confirmed by the neurologist. To ensure that we captured peak medication effects in the ON-state, all patients arrived in OFF-state to the clinic. This was confirmed by the neurologist. When the session was ON-state, the patient took his/her scheduled L-DOPA dose and the evaluation began after both the participant and neurologist confirmed the ON-state (state ON/OFF questioning was performed every 0.5 h until ON or 1.5 h post-dose, whatever was earlier). If the first session was in ON-state, the second session (OFF-state) began between 0.5 to 1 h before their next scheduled L-DOPA dose the same day or up to 14 days later.

In the first speech task, participants described the “Cookie Theft” picture from the Boston Diagnostic Aphasia Exam^64^ and a second, similar picture, the “Lightbulb Changing”^65^ (both pictures are shown in Supplementary Fig. 1). Participants were asked to provide a verbal description of the picture. The “Cookie Theft” picture was presented to all participants in session 1 and the “Lightbulb Changing” picture in session 2. The objective of this task was to evaluate cognitive skills and communication ability, as well as to check for changes in action verb use^33–39^^,42^. The second task was reverse counting, a modification of the classic test for mental state evaluation^66,67^ where participants count backward by three, starting from a different (experimenter-provided) number in each session, to maintain the level of cognitive difficulty. This cognitive assessment test evaluates concentration in the participant. The third speech task was a diadochokinetic rate test widely used for assessing dysarthria^42,68^ to measure speech production. In this test, participants were asked to pronounce the syllable sequence “pa-ta-ka” as rapidly as possible for 10 s.

### Data acquisition

To record the speech tasks, participant wore a Shure SM10A, a head-mounted, low-impedance, dynamic cardioid microphone. Audacity software^69^ was used to record the speech task using 16-bits at 44.1 kHz. All audio recordings were saved in the uncompressed ‘.wav’ format.

### Feature extraction

Processing of the speech recordings was performed using Python^70^ and Praat^71,72^. Acoustic and prosodic (speech tempo) features were computed in all three speech tasks, and semantic features were computed for picture description, as explained below.

Speech production in humans is the result of modulating the source of sound energy that comes from the larynx with different parts of the vocal tract (e.g., the oral cavity). Speech degradation such as the one presented in Parkinson’s disease is usually a consequence of anomalies in the functionality of the parts of the vocal tract (e.g. imprecise articulation) or in the source (e.g., breathy voice). Cepstral analysis is useful for speech analysis as it can separate the sound source from its modulation. MFCCs is a technique that not only incorporates cepstral analysis, but also uses a non-linear scale (Mel scale) that approximates the human auditory system’s response. Due to these advantages, MFCCs have been used in speaker identification methods, speech quality assessment^73^, and in classification of neurological diseases^23,26,74^ with great accuracy. Thirteen MFCCs were calculated using the “python-speech-features” package^75^. Following common practice^76^, the first coefficient was replaced by the log of the total frame energy in order to analyze the overall energy in the speech. To calculate the coefficients, a window size of 25 milliseconds (ms) and window overlap of 10 ms were used, and pauses were automatically removed from the recording. A pause was defined by a silence threshold of −25 dB and minimum duration of 100 ms^77^. To represent the distribution of each coefficient, we computed 10 statistical descriptors: mean (mn), variance (vn), kurtosis (kur), skewness (sk), mode (mod), percentiles 10th (pct10), 25th (pct25), 50th (pct50), 75th (pct75), and 90th (pct90). The rationale for using these statistical descriptors is that most of these distributions are not Gaussian, and therefore mean and variance do not fully characterize them. For any distribution, kurtosis is a measure of how “wide” or “narrow” it is, skewness is a measure of its asymmetry, the 50th percentile (or median) is a robust (to outliers) measure of the central tendency of the distribution, and similarly the 10th and 90th percentiles are robust versions of the minimum and maximum. These 130 features (10 descriptors for each of 13 coefficient distributions) were calculated on all speech tasks.

To characterize speech tempo, a prosodic feature, we used nuclei syllable (NS)^78^. This feature estimates the temporal location of syllables within the speech stream. This analysis detects individual syllables in speech by identifying peaks in intensity (i.e., loudness) that are preceded and followed by dips in intensity. We then computed the elapsed time between syllables in the speech recording both with and without pauses removed. To represent the distribution of syllable duration, we computed 8 statistical descriptors (percentiles 10 and 90, mode, mean, variance, skewness, kurtosis plus interquartile range—IQR, a measure of variability).

As the picture description task elicited narrative speech, we also analyzed the semantic content of the description (semantic features; SF). The theory of embodied cognition posits that the motor (action) and sensory (perception) systems influence overall cognitive processing, and in particular, linguistic processing of words that are related to motor and sensory processes, and that they activate the same brain networks. Thus, the strong form of the theory predicts that people who have impaired movement abilities, such as those with PD, should show deficits in linguistic processing of words related to actions and motor activities^79,80^. For this reason, we calculated semantic similarity to evaluate the relationship between the descriptions of the pictures by the participants and action or non-action words. To compute the similarity, we first isolated the nouns and verbs from the manually-transcribed recordings using the Stanford parser^81^. Based on the previous literature^33–39,42^, the following seed words were chosen as action or non-action base words for calculating semantic distance: *action*, *act*, *move*, *play*, *energetic*, *inaction*, *sleep*, *rest*, *sit* and *wait*. Next, we obtained a numerical representation of all the words using Global Vectors for word representations (GloVe)^82,83^. Finally, similarity distance (where a larger value indicates “more similar” not “farther away”) was computed between each verb and noun spoken by the participant and each of the seed words. To represent the distribution obtained for each seed word, the following statistical descriptors were calculated from the distances of the participant’s words: median, 10th percentile, 90th percentile, skewness, kurtosis, IQR (Inter Quartile Range). The total number of words (nw) was also computed in the analysis.

### Statistical analysis

To evaluate whether these speech production features were sensitive to the differences between the two medication states, we performed two-sample paired *t*-tests for each feature, comparing their values across participants in the “ON” versus “OFF” state. To investigate how the features interacted in each medication state, we also computed the partial correlations among the top features for each speech task. Partial correlation captured the pattern of covariation between a pair of features by removing the effect of the other analyzed features.

### Classification

We evaluated whether our features could differentiate one medication state from another by applying four general-purpose classifiers: elastic net (EN), logistic regression (LR) with l1-norm regularization, naive Bayes (NB) and random forest (RF). Since it has been demonstrated by Rusz et al.^84^ that age and gender can bias the results in PD vs. control classification tasks, we focused our analysis on subject-based changes. For this, we calculated the difference of the speech features between the two states (“ON”/”OFF”) for each participant. Features were standardized (mean = 0 and standard deviation = 1) before being input to the classifiers. We used 10-fold cross-validation, leaving entire participants out in the test folds. We chose a fixed number of features to provide the classifiers, selecting the top-ranked 5 features. Other parameters, including those specific to a classifier type, were selected through a double-nested approach to avoid over-fitting. To determine which features were the highest-ranked and should be included in the classifier (feature selection), all features were rank-ordered based on the p-values from the paired t-tests on the training folds to order the set by how well each feature individually discriminated between the two classes (“ON” and “OFF”). After feature selection, we ran each of the four classifiers using the 5 top-ranked features, for each category of features (NS only, MFCCs only, MFCCs + SF, etc., further details below). Finally, accuracy rates were calculated over 50 instantiations of the 10-fold partition; we provide mean and standard deviation and report the best classifier. For comparison, we also run the same classifiers using all of the features (see Supplementary Table 2).

### Reporting summary

Further information on research design is available in the Nature Research Reporting Summary linked to this article.

## Supplementary information

Supplementary Material

reporting summary

## Data Availability

The data used in this study are available on request from the corresponding author R.N. and agreement with Tufts University through B.K.H. The data are not publicly available due to voice being potentially identifiable, which could compromise research participant privacy.

## Code availability

Our methodology is implemented using open-source libraries from Python and Praat only. Here, we specify the names and versions of the libraries used for each section of our approach. MFCC features were extracted using python-speech-features v5.0 library. For prosody, specifically nuclei syllable, information was obtained using SyllableNuclei v.Sep2010 Praat script^78^ and pauses were removed using PraatVocalToolKit^85^. For semantic features, the transcripts were processed using the Stanford Parser^81^ lexparser v.2017 and the semantic embedding was obtained using GloVe v1.2^82,83^. All statistical analyses were performed using numpy v.1.12.0 and scipy v.0.18.1 python libraries. Finally, for classification, we trained our models using scikit-learn 0.18.1 and sklearn-contrib-lightning v0.5.0 python libraries. For each algorithm, we fixed the values of some parameters and selected the optimal values of other ones from a pool of values using a cross validation approach. For random forests, we used a fixed number of trees (20) and we optimized the maximum depth from these values: [2, 3, 5, 7, 10, 16]. Similarly, logistic regression with l1-norm regularization had a fixed tradeoff parameter of C = 1, and we optimized the amount of regularization from this set of values: [1E−5, 2.5E−4, 6.3E−3, 1.6E−1,4, 1E2]. For elastic net, we optimized the amount of regularization and the l1/l2 ratio using the set of values [1E−4, 1E−2, 1, 1E2, 1E4] and [0.001, 0.01, 0.1, 0.5, 0.9], respectively. Finally, naive Bayes was used with the default parameters.
