# Supplementary material for: Speech-based characterization of dopamine replacement therapy in people with Parkinson’s disease
Source: NPJ Parkinsons Dis. 2020 Jun 12;6:12. doi: 10.1038/s41531-020-0113-5 (PMC7293295; doi:10.1038/s41531-020-0113-5)
Supplement: Supplementary file 1 — Supplementary Material [file 41531_2020_113_MOESM1_ESM.pdf]

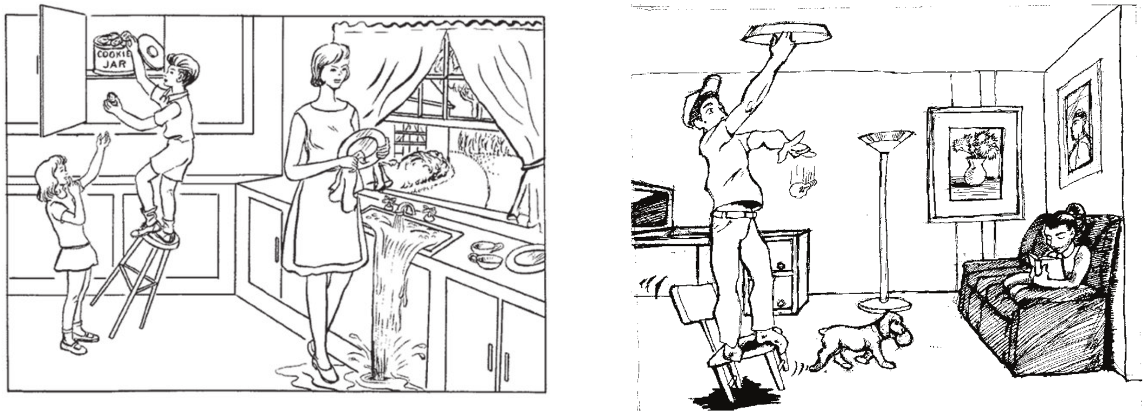

Supplementary Figure 1: Pictures used as stimuli for Picture Description task. Picture to the left[1] (From the Boston Diagnostic Aphasia Examination – Third Edition by Harold Goodglass, Edith Kaplan and Barbara Barresi. Used with permission of PRO-ED, Inc.) is described in first visit while picture to the right[2] (From Marshall & Wright, Am. J. Speech-Language Pathol. (2007). Used with permission of ASHA) is described during the second visit.

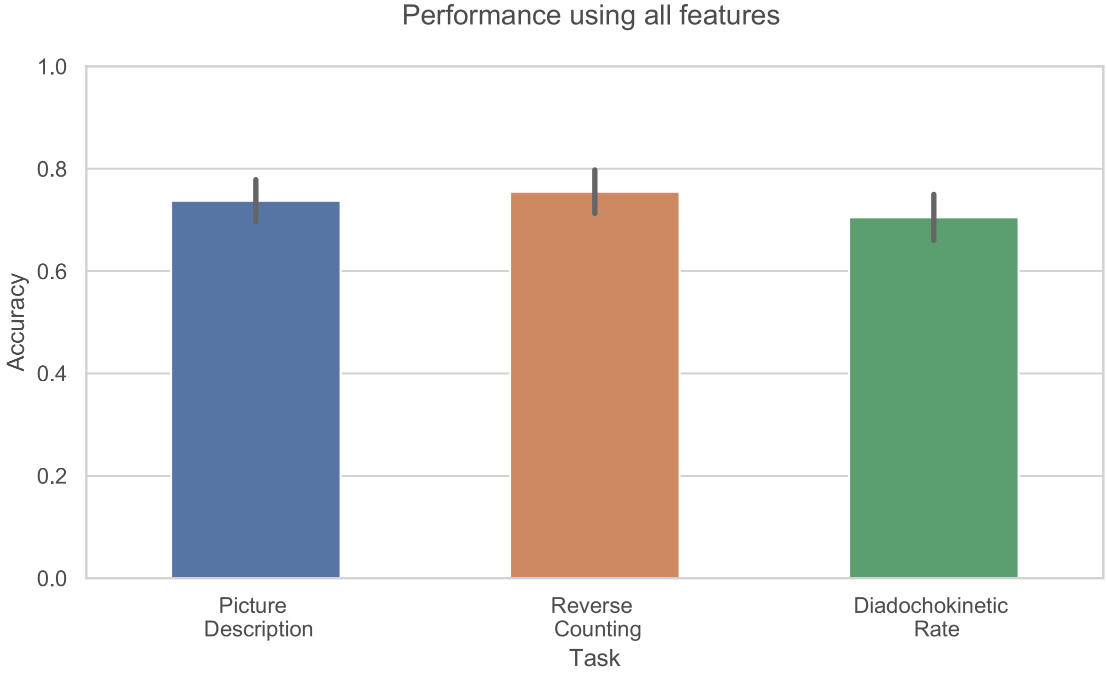

Supplementary Figure 2: Classification performance for each task without using feature selection. Classification accuracy when using all the features, using 10-fold (by subject) cross validation. Bars show mean of 50 runs, vertical lines denote standard deviation. Results surpass chance probability.

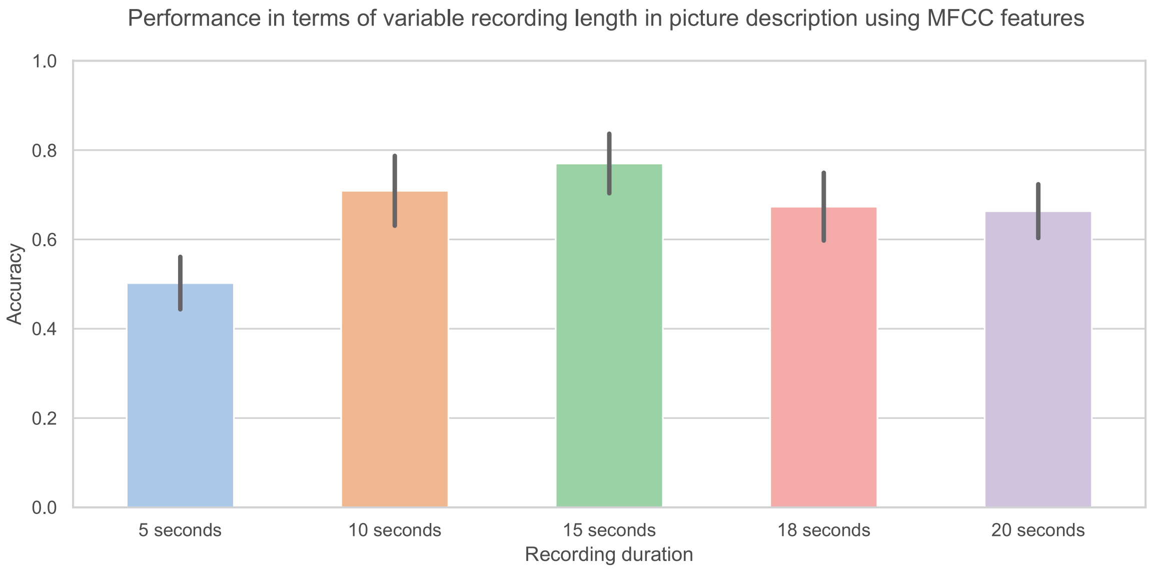

Supplementary Figure 3: Audio Duration Impact on Classification Performance. ON/OFF Classification accuracy for audio clips of 5, 10, 15, 18 and 20 seconds. Only 18 subjects were used for this analysis since we used recordings of 20 or more seconds after removing pauses. The features used in this analysis were MFCCs given the independence of context. Bars were created based on 10-fold cross validation on 50 runs, depicting mean and standard deviation. Mean accuracy rate is stable around 0.70 for different recording lengths except for 5 seconds.

Supplementary Table 1. Medications taken by participants.

SubjectCode	Medication	DoseFreeTex	Schedule
3001	sinemet ER	50/200	1 tab at bedtime
3001	vitamin d	1000	daily
3001	pravastatin	20	daily
3001	ecotrin	81	daily
3001	sinemet	25/100	3 tabs twice a day
3001	atenolol	100	daily
3002	comtan	200	tid
3002	norvasc	10	daily
3002	tamsulosin	0.4	daily
3002	pramipexole	0.5	tid
3002	lisinopril	5	daily
3002	pravachol	20	daily
3002	aspirin	325	daily
3002	sinemet	25/100	2 tabs tid
3002	amantadine	100	2 tabs bid
3002	gabapentin	300	qhs
3002	prilosec	20	daily
3003	pravachol	20	daily
3003	sinemet	25/100	3 tabs TID
3003	trazodone	50	qhs
3003	paroxetine	20	daily
3003	asacol	400	bid
3004	omeprazole	20	daily
3004	isradipine	2.5	daily
3004	aspirin	81	daily
3004	sinemet	25/100	TID
3004	citalopram	20	daily
3004	hydrochlorot	12.5	daily
3005	simvastatin	20	daily
3005	warfarin	4	daily
3005	sinemet	25/100	TID
3005	azilect	1	daily
3005	lisinopril	20	daily
3005	hydrochlorth	12.5	daily
3005	pramipexole	0.5	at bedtime
3006	vitamin D3	1000	daily
3006	calcium-vitai	600-400	two tabs daily
3006	balsalazide	750	TID
3006	metformin	1000	BID
3006	atorvastatin	40	daily
3006	sinemet	25/100	2 tabs TID
3006	fludrocortiso	0.1	daily
3006	aspirin	81	daily
3007	gabapentin	800	1 tab daily
3007	clonazepam	0.5	at bedtime
3007	amantadine	100	2 caps TID
3007	ropinirole	2	1 tab at bedtime
3007	Azilect	1	1 tab daily
3007	Rytary	48.75/195	3 tabs TID
3007	ropinirole ER	12	1 tab daily
3008	comtan	200	five times a day
3008	pramipexole	4.5	daily
3008	vitamin D	1000	daily
3008	amantadine	100	four times a day
3008	sinemet	25/100	3 tabs five times a

SubjectCode	Medication	DoseFreeTex	Schedule
3009	multivitamin	n/a	daily
3009	calcium	600	daily
3009	tolterodine	4	daily
3009	Neupro	3	daily
3009	Azilect	1	daily
3009	Sinemet ER	25/100	1 tab TID
3009	hydrochlorth	12.5	daily
3009	crestor	5	daily
3010	sinemet	25/100	TID
3010	rasagiline	1	daily
3010	simvastatin	20	daily
3011	carbidopa/le	25/100	5 times
3011	selegiline	5	bid
3011	neupro	2	24hr patch
3012	parcopa	25/100	daily as needed
3012	simvastatin	40	daily
3012	sinemet	25/100	2 tabs TID
3012	mirapex	0.25	at bedtime
3012	Aspirin	81	daily
3012	levothyroxine	0.05	daily
3012	buproprion	150	daily
3012	hydrochlorthi	25	daily
3012	protonix	20	daily
3012	finasteride	5	daily
3013	sinemet	25/100	twice a day
3013	sinemet CR	50/200	at bedtime
3013	omeprazole	20	2 caps daily
3013	atorvastatin	10	daily
3013	diltiazem	300	daily
3013	aspirin	81	daily
3013	pramipexole	1.5	three times a day
3015	tumeric-curc	500	daily
3015	aspirin	81	daily
3015	Neupro	4	daily
3015	ubiquinol	300	daily
3015	vitamin D-3	5000	daily
3015	sinemet	25/100	TID
3015	glutathione	150	daily
3015	omega-3	450	daily
3015	Artane	2	TID
3016	lipitor	40	daily
3016	diovan	80	daily
3016	hydrochlorthi	25	daily
3016	entacapone	200	twice daily
3016	sinemet	25/100	twice daily
3016	rasagiline	1	daily
3017	sinemet ER	25/100mf	qhs
3017	dulera	5mcg	qday
3017	proventil	1	prn
3017	buproprion	150	qday
3017	inosine	500mg	qday
3017	sinemet	25/100	2 pills tid
3018	claritin	unknown	daily
3018	Sinemet	25/100	0.5 tab TID
3018	magnesium	unknown	daily
3018	calcium-vitai	600-400	daily
3018	ropinirole	2	TID
3018	simvastatin	40	daily
3018	aspirin	81	daily

SubjectCo	Medication	DoseFreeTex	Schedule
3019	sinemet	25/100	2 tabs TID
3019	vitamin		
3019	lisinopril		
3019	metoprolol		
3020	meripex er	3	qam
3020	azilect	1	qday
3020	sinemet .5 ta	25/100	TID
3021	premarin	0.625	twice weekly
3021	Sinemet	25/100	three times a day
3021	Rasagiline	1	daily
3021	vitamin E	400	twice daily
3021	aspirin	81	daily
3021	atenolol	25	daily
3021	fluticasone p	50	daily
3021	CoQ10	400	three times daily
3021	atorvastatin	40	daily
3021	meclizine	25	as needed
3021	vitamin D3	1000	twice daily
3021	ropinirole	4	three times daily
3021	B12 injectior	1000	monthly
3022	crestor	10	daily
3022	prednisone	10	daily
3022	sinemet	25/100	1.5 tabs qid
3022	amiodarone	200	daily
3022	lisinopril-hyd	20-25	daily
3022	metoprolol	50	twice daily
3022	selegiline	5	daily
3023	simvastatin	20	daily
3023	zonisamide	25	daily
3023	bupropion	75	twice a day
3023	sinemet	25/100	six times a day
3023	Nuvigil	200	0.5 tab daily
3023	Aziledt	1	daily
3023	famotidine	20	daily
3023	keppra	500	twice a day
3023	Lovenox	40	daily
3023	sinemet CR	25/100	at bedtime
3023	neupro	8	daily
3024	carbidopa-le	25-100	TID
3024	omega-3 fat	1000	QD
3024	amantadine	100	BID
3024	omeprazole	20	QD
3024	aspirin	81	QD
3024	clonazepam	0.5	QD
3024	cholecalcifer	2000	QD
3024	folic acid	800	QD
3024	multivitamins		QD
3024	simvastatin	20	QD
3024	sildenafil citr	20	QD
3025	trihexyphend	2	daily
3025	diovan		daily
3025	hydrochlorth	25	daily
3025	levothyroxine	2	daily
3025	sinemet	25/100	four times a day
3025	vitamin D3	2000	daily

Supplementary Table 2. Classification performance**.** Performance achieved in each task for the 3 different feature sets. Only the classifiers with highest accuracy value are shown. Accuracy is computed as the average (±s.d.) of 50 runs with 10-fold cross-validation. The highest accuracy rate is obtained for picture description using 5 top-rank features. MFCC features are relevant for achieving good performance in the different speech tasks.

**Speech Task (# patients)**	**Features**	**Classifiers**	**Top 5 features**	**All features**
Picture description (25 patients)	NS	RF	0.61±0.05	0.58±0.04
	SF	RF	0.77±0.04	0.63±0.05
	NS + SF	EN	0.79±0.07	0.65±0.05
	MFCC	EN	0.54±0.08	0.73±0.07
	MFCC + NS	EN	0.50±0.06	0.74±0.04
	MFCC + SF	LR-l1	0.89±0.06	0.64±0.07
	MFCC + SF + NS	LR-l1	0.89±0.05	0.63±0.08
Reverse counting (25 patients)	NS	RF	0.41±0.07	0.51±0.05
	MFCC	NB	0.84±0.02	0.64±0.06
	MFCC + NS	RF	0.79±0.03	0.76±0.04
Diadochokinetic rate (24 patients)	NS	LR-l1	0.53±0.06	0.70±0.05
	MFCC	NB	0.60±0.07	0.56±0.06
	MFCC + NS	NB	0.58±0.06	0.58±0.05

Supplementary References:

1. Goodglass, H., Kaplan, E. & Barresi, B. *The assessment of aphasia and related disorders*. (Lippincott Williams & Wilkins, 2001).
2. Marshall, R. C. & Wright, H. H. Developing a clinician-friendly aphasia test. *Am. J. Speech-Language Pathol.* (2007).
